# Supplementary material for: Modulation of Kv Channel Gating by Light-Controlled Membrane Thickness
Source: Biomolecules. 2025 May 21;15(5):744. doi: 10.3390/biom15050744 (PMC12109740; doi:10.3390/biom15050744)
Supplement: Supplementary file 1 [file biomolecules-15-00744-s001.zip › biomolecules-3624092-supplementary.pdf]

# Supplementary Information to

# Modulation of Kv Channel Gating by Light-Controlled Membrane Thickness

Rohit Yadav[1,†], Juergen Pfeffermann[1,†], Nikolaus Goessweiner-Mohr[1], Toma Glasnov[2], Sergey A. Akimov[3], Peter Pohl[1,*]

[1]Institute of Biophysics, Johannes Kepler University Linz, Linz, Austria

[2]Institute of Chemistry, Karl-Franzens-University, Graz, Austria

[3]Frumkin Institute of Physical Chemistry and Electrochemistry,

Russian Academy of Sciences, Moscow, Russia

[†] These authors contributed equally to this work.

*Correspondence to: peter.pohl@jku.at

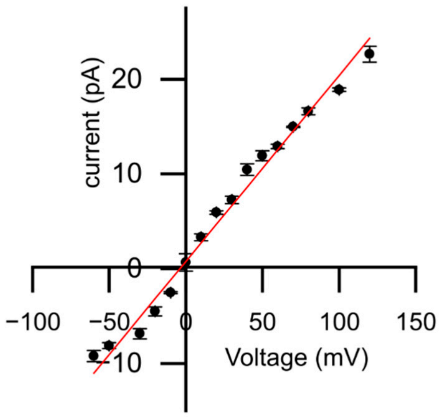


**Figure S1.** Single-channel IV curve, Single-channel current of KvAP as a function of membrane voltage, recorded in photoswitchable PLBs folded from PLE with 20 m% trans-OptoDArG in 150 mM KCl, 10 mM HEPES, pH 7.4.

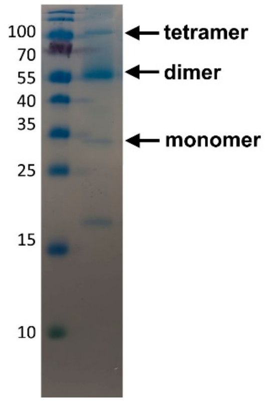


**Figure S2.** SDS-PAGE image.

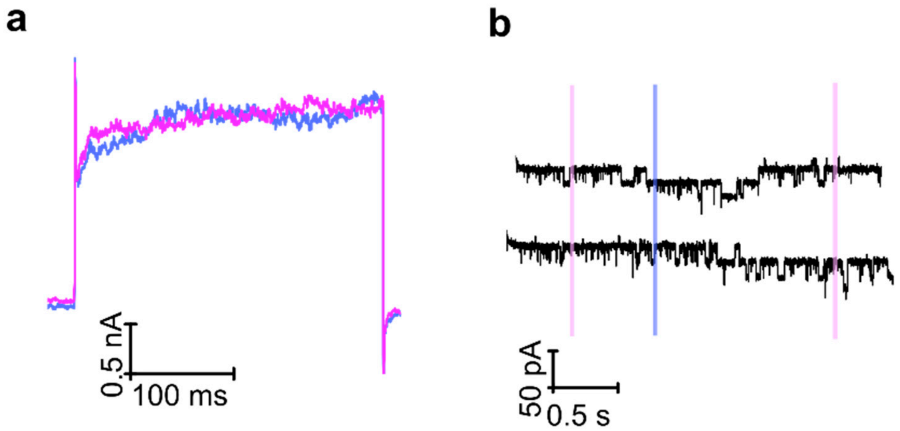


**Figure S3.** Photoswitching of KvAP in the absence of OptoDArG (control). (a) Ensemble KvAP currents recorded during voltage steps from −80 mV to +100 mV (holding potential −100 mV) in a PLB composed of E. coli lipid and reconstituted with KvAP. The recording solution contained 150 mM KCl and 10 mM HEPES (pH 7.4). Currents were recorded under continuous UV (magenta) and blue laser (blue) illumination. (b) Single-channel recordings of KvAP at +120 mV with exposure to 40 ms pulses of UV (magenta bars) and blue (blue bars) laser light.

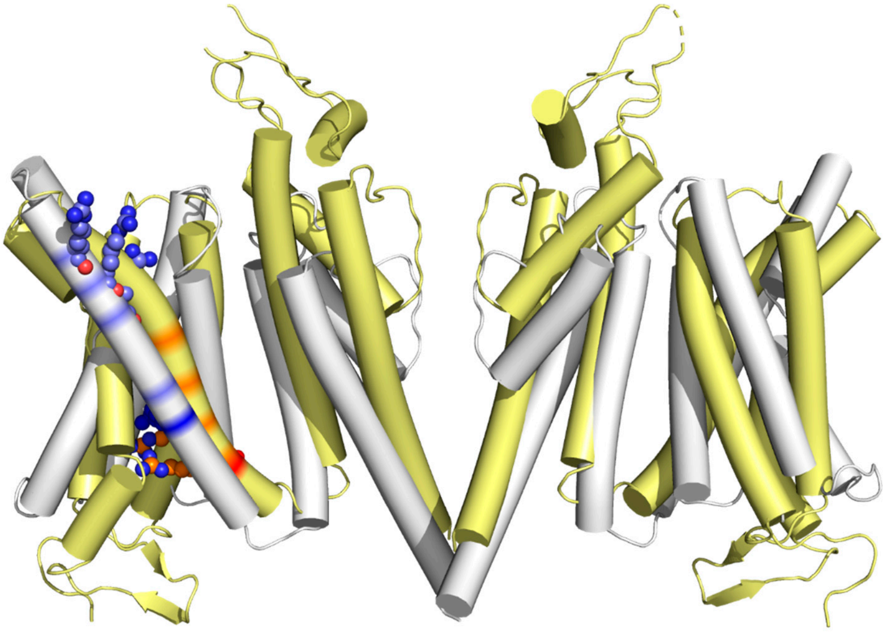

**Figure S4.** Alignment of the channels Eag Kv (yellow, PDB: 8EOW) and KvAP (white, PDB: 6UWM). Despite very low sequence homology, alignment of the structures in PyMol led to an acceptable fit. The four important Arg residues responsible for voltage sensing in KvAP [1] are highlighted in light blue. A close Lys (dark blue) provides a 5th positive charge. Similarly, Eag Kv possesses 4 conserved Arg (orange) and a conserved Lys (red) next to the lowest Arg position.

**Supplementary Note:** Estimate of Electrostatic Energy Change Due to Lipid Displacement

The double electrical layer near a charged membrane can be modeled as a planar capacitor with specific capacitance $C_0$ [2]. The energy of the capacitor is:

$$E_0 = \frac{Q^2}{2C_0S_0} \quad \text{(S1)}$$

where Q is the total electric charge and $S_0$ is the area of the membrane.

Upon pulling $N$ = 8 lipid molecules (in a tetrameric channel, each monomer pulls two lipid molecules) up by a height $h \approx 0.5$ nm each, the area of the double layer should increase by the area $\Delta S$. This increase in area can be estimated under the assumption that an initially planar double layer of area $S_0 = \pi r^2$ near each molecule transforms into a segment of a sphere with height h. The area $A_s$ of such a segment is equal to:

$$A_s = 2\pi R_s h \quad \text{(S2)}$$

where $R_s$ is the radius of the sphere. Since

$$R_s = \frac{r^2 + h^2}{2h} \quad \text{(S3)}$$

we find for $\Delta S$:

$$\Delta S = A_s - S_0 = \pi h^2 \quad \text{(S4)}$$

The corresponding change in the electrostatic energy can thus be estimated as:

$$\Delta E = \frac{Q^2}{[2C_0(S_0 + \Delta S)]} - \frac{Q^2}{2C_0S_0} = \frac{Q^2}{2C_0S_0}\left[\frac{1}{(1 + \Delta S/S_0)} - 1\right] \quad \text{(S5)}$$

Given that $\Delta S \ll S_0$, we can approximate:

$$\Delta E \approx \frac{Q^2}{S_0^2}\frac{\Delta S}{2C_0} \quad \text{(S6)}$$

Let $\sigma = Q / S_0$ — the surface density of electric charge of the lipid monolayer. Then:

$$\Delta E \approx \frac{\sigma^2 \Delta S}{2C_0} \quad \text{(S7)}$$

The specific capacitance of the double electric layer is:

$$C_0 = \frac{\varepsilon\varepsilon_0}{\lambda_D} = \frac{\varepsilon\varepsilon_0}{\sqrt{\frac{\varepsilon\varepsilon_0 RT}{2F^2 I}}} = \sqrt{\frac{2\varepsilon\varepsilon_0 F^2 I}{RT}} \quad \text{(S8)}$$

where $\lambda_D$ is the Debye length of the electrolyte; *R* is the gas constant; *T* is the absolute temperature; *F* is the Faraday constant; $\varepsilon$ and $\varepsilon_0$ are the dielectric permittivities of the electrolyte and vacuum, respectively; *I* is the concentration of the binary electrolyte.

For a 28% mole fraction of charged lipids in the membrane, $\sigma \approx 0.28 \cdot e / a_0 \approx 0.064$ C/m$^2$, where $a_0 \approx 0.7$ nm$^2$ is the area per lipid molecule and $e = 1.6 \times 10^{-19}$ C is the elementary charge. At physiological conditions (*I* = 150 mM in the bulk), we estimate a $K^+$ concentration of 482 mM and a $Cl^-$ concentration of 47 mM close to the membrane for a membrane potential of –30 mV. Accordingly, we find $C_0 \approx 1.6$ F/m$^2$. Substituting $\Delta S$, $C_0$, and $\sigma$ into Eq. (5) yields –0.23 kT, where kT ≈ $4.1 \times 10^{-21}$ J.

By increasing the bulk ionic strength 10-fold, we obtain a $K^+$ concentration of 1822 mM and a $Cl^-$ concentration of 1235 mM close to the membrane for a membrane potential of –5 mV. $C_0 \approx 2.8$ F/m$^2$, and consequently $\Delta E \approx -0.13 \cdot kT$. By decreasing the bulk ionic strength 10-fold (with respect to physiological conditions), we obtain a $K^+$ concentration of 155 mM and a $Cl^-$ concentration of 1.5 mM close to the membrane for a membrane potential of –60 mV, $C_0 \approx 0.89$ F/m$^2$, and consequently $\Delta E \approx -0.42 \cdot kT$.

Thus, $\Delta E$ varies by about 0.29·kT upon a 100-fold variation in bulk ionic strength, supporting the assumption in the main manuscript that this electrostatic effect is too small to be captured in our experiments. It is also more than an order of magnitude smaller than the free energy required for gating charge movement [3].

**References**

1. Ruta, V.; Jiang, Y.; Lee, A.; Chen, J.; MacKinnon, R. Functional analysis of an archaebacterial voltage-dependent K+ channel. *Nature* **2003**, *422*, 180-185, doi:10.1038/nature01473.

2. Batishchev, O., V; Shilova, L.; Kachala, M., V; Tashkin, V.; Sokolov, V.; Fedorova, N., V; Baratova, L.; Knyazev, D.; Zimmerberg, J.; Chizmadzhev, Y. pH-Dependent Formation and Disintegration of the Influenza A Virus Protein Scaffold To Provide Tension for Membrane Fusion. *J. Virol.* **2016**, *90*, 575-585.

3. Islas, L.D.; Sigworth, F.J. Electrostatics and the gating pore of Shaker potassium channels. *J. Gen. Physiol.* **2001**, *117*, 69.
